# Optimizing household survey methods to monitor the Sustainable Development Goals targets 6.1 and 6.2 on drinking water, sanitation and hygiene: A mixed-methods field-test in Belize

**DOI:** 10.1371/journal.pone.0189089

**Published:** 2017-12-07

**Authors:** Shane M. Khan, Robert E. S. Bain, Karsten Lunze, Turgay Unalan, Bo Beshanski-Pedersen, Tom Slaymaker, Richard Johnston, Attila Hancioglu

**Affiliations:** 1 Division of Data, Research and Policy, UNICEF, New York City, New York, United States of America; 2 School of Medicine, Boston University, Boston, Massachusetts, United States of America; 3 Department of Public Health, Environmental and Social Determinants of Health, World Health Organization, Geneva, Switzerland; University of Vermont, UNITED STATES

## Abstract

**Background:**

The Sustainable Development Goals (SDGs) require household survey programmes such as the UNICEF-supported Multiple Indicator Cluster Surveys (MICS) to enhance data collection to cover new indicators. This study aims to evaluated methods for assessing water quality, water availability, emptying of sanitation facilities, menstrual hygiene management and the acceptability of water quality testing in households which are key to monitoring SDG targets 6.1 and 6.2 on drinking Water, Sanitation and Hygiene (WASH) and emerging issues.

**Methods:**

As part of a MICS field test, we interviewed 429 households and 267 women age 15–49 in Stann Creek, Belize in a split-sample experiment. In a concurrent qualitative component, we conducted focus groups with interviewers and cognitive interviews with respondents during and immediately following questionnaire administration in the field to explore their question comprehension and response processes.

**Findings:**

About 88% of respondents agreed to water quality testing but also desired test results, given the potential implications for their own health. *Escherichia coli* was present in 36% of drinking water collected at the source, and in 47% of samples consumed in the household. Both questions on water availability necessitated probing by interviewers. About one quarter of households reported emptying of pit latrines and septic tanks, though one-quarter could not provide an answer to the question. Asking questions on menstrual hygiene was acceptable to respondents, but required some clarification and probing.

**Conclusions:**

In the context of Belize, this study confirmed the feasibility of collecting information on the availability and quality of drinking water, emptying of sanitation facilities and menstrual hygiene in a multi-purpose household survey, indicating specific areas to improve question formulation and field protocols. Improvements have been incorporated into the latest round of MICS surveys which will be a major source of national data for monitoring of SDG targets for drinking water, sanitation and hygiene and emerging issues for WASH sector programming.

## Introduction

The adoption of the Sustainable Development Goals (SDGs) in September 2015 set out an ambitious new agenda for global development. Whereas the Millennium Development Goals (MDGs) had 8 goals and 21 targets, the SDGs have 17 goals and 169 targets [1,2]. The unprecedented increase in the amount of data needed for SDG monitoring has inspired calls for a ‘data revolution’, which necessitates innovative approaches to data collection, analysis and use [3]. The SDGs have also drawn attention to the need to expand the envelope of options to collect monitoring data but also the critical role that key data sources such as household surveys will continue to play in tracking progress and monitoring inequalities.

Household surveys are a rich source of data on major development goals such as the MDGs, and for sectoral data on water, sanitation and hygiene (WASH). Indeed, in the final assessment of the MDGs, 84 percent of data on WASH indicators came from household surveys [4]. The UNICEF-supported Multiple Indicator Cluster Surveys (MICS) is a key global data source and was established in the mid-1990s to support countries to collect representative data on the situation of children and women [5]. Close to 300 MICS surveys have been conducted in over 100 countries and MICS is entering the sixth round of surveys (MICS6). Without doubt, MICS and other survey programmes, such as the USAID-supported Demographic and Health Surveys (DHS) and the World Bank-supported Living Standards Measurement Study (LSMS) which routinely collect data on WASH, will continue to be instrumental in monitoring progress against national and international development goals for WASH and other sectors [6].

During the MDG era, the WHO/UNICEF Joint Monitoring Programme for Water Supply and Sanitation (JMP), which sets global standards for monitoring progress on water, sanitation and hygiene, estimated the use of ‘improved’ drinking water sources and sanitation facilities based on national data sources. Improved drinking water sources are those designed to protect against contamination, especially faecal matter, while improved sanitation facilities are those designed to safely separate excreta from human contact [7]. These terms have been widely used with the known limitation that improved facilities are not always ‘safe’. For example, water from a piped source or borehole, which is classified as ‘improved’, may still contain faecal contamination [8] while use of improved sanitation facilities may not prevent sewage and faecal sludge from being discharged into the environment without treatment [9]. The new global indicators for SDG targets 6.1 and 6.2 address these concerns by setting a higher benchmark of ‘safely managed’ drinking water and sanitation services.

‘Safely managed drinking water’ builds on the established indicator ‘use of an improved drinking water source’ and introduces three additional criteria: 1) the improved source is located on premises, 2) water should be available when needed, and 3) water supplied should be free from faecal and priority chemical contamination. While previous MICS surveys have included information on the type and location of drinking water sources used by households, questions on water availability and quality are new. *Escherichia coli* (*E*.*coli*) is the recommended indicator of faecal contamination which is a major concern for human health in all countries [10]. Evidence from a systematic review suggests that 1.8 billion people use a source of drinking water contaminated with faecal indicator bacteria [11].

‘Safely managed sanitation’ also builds on the established ‘improved/unimproved’ facility classification used in MICS and other surveys and addresses the subsequent management of excreta including safe treatment and disposal of excreta, which is an emerging issue in varied settings [12,13].

SDG Target 6.2 also addresses hygiene. The global SDG indicator focusses on access to a handwashing facility with water and soap at home, as a proxy for individual hygiene practices [14,15]. This indicator was developed by the MICS programme and has been used in surveys supported by MICS and DHS since 2009. Another emerging priority issue for the WASH community is management of menstruation. The current literature suggests that menstrual hygiene management (MHM) can be a challenge for women and girls, especially in low and middle-income settings though there have been few large-scale studies of the impacts on health, education and gender equality [16–19]. As this is a potential area for large-scale measurement, there is a need to test new questions on this topic that can be recommended to countries.

While the new SDG indicators for WASH build on established indicators, thereby providing continuity for monitoring, new elements place considerable demands on national household surveys to develop new data collection methodologies that respond to emerging data needs. The MICS survey programme, as part of its ongoing work to ensure that appropriate tools are available to countries for collecting household survey data, periodically updates all survey tools, including standard questionnaire modules and survey protocols. We designed a mixed-methods field test of MICS tools in Belize to assess the suitability and performance of new SDG WASH indicators for the MICS surveys. The objectives of this study are to examine the process of testing drinking water, both in the household and at the source, for *E*. *coli* as part of a MICS survey, assess a minimum set of new questions on the availability of drinking water, test respondent’s knowledge about faecal sludge management and evaluate questions on menstrual hygiene. The findings of this study will inform the uptake of questions and protocols into the MICS surveys and help shape WHO/UNICEF JMP recommendations for enhanced WASH monitoring.

## Data and methods

This mixed-methods study evaluated questions and protocols implemented in a MICS field test in Stann Creek district, Belize from November to December 2015. Data were collected using quantitative and qualitative methods, which is a widely recommended practice in survey-development and questionnaire evaluation [20]. Prior to each element of data collection, interviewers explained the informed consent process, providing the purpose and content of the study and explaining that participation was optional and could be revoked by the participants at any time during the process. Participants then provided verbal consent to begin the interview or declined to participate. No personal identifiers were documented. The Statistical Institute of Belize (SIB) as the national authority on household data collection provided ethical oversight, reviewed all data collection protocols and approved all study protocols. Belize was selected for the field test as the country provides wide variation in respondents’ background such as education, level of rurality and wealth, which are desirable when testing questions across different domains and which reflects the variation which exists in the different countries that implement MICS. Further, as SIB had recently completed a national MICS survey, the field test used the same field workers for the field test, being able to take advantage of their intimate knowledge of the questions and protocols.

### Quantitative approach

#### Household survey with split-sample design

A representative household survey of Stann Creek district, Belize was implemented using a 2-stage probability design. In the first stage, 20 census enumeration areas were randomly selected and in the second stage, 30 households in each area were selected using systematic sampling. Households were numbered sequentially from 1 to 30 and split into two samples based on even or odd numbers. These two samples allowed us to compare alternative formulations of selected questions on an experimental basis. The household survey collected quantitative data on newly developed questions using tablet computers. New areas of measurement included water quality, water availability, menstrual hygiene management, and emptying of septic tanks and pit latrines. Two teams of five female interviewers, employed by SIB, were trained by the MICS and WHO/UNICEF JMP teams with support from UNICEF Belize and the national ministry of health and the ministry of human development and social transformation. These field teams implemented the household survey under the supervision of two field supervisors and the MICS team. The household survey had 5 questionnaires. The first was a household questionnaire, followed by a questionnaire for individual women, a water quality testing questionnaire, a child functioning questionnaire and finally, a questionnaire for under-fives. The field-test was not dedicated to WASH-only topics but also contained a number of new topics and questions for testing. Topics covered a wide range such as child function, victimization, changes to approaches to testing salt for iodization, dietary recall and social transfers. Sample weights were designed to take into account non-response (as per usual MICS practice) and used in this analysis. The response rates were 91 percent for households, 67 percent for individual women and 87 percent for water quality testing. Unless specified, the same questions were asked of both samples.

Regarding drinking water, interviewers asked household questionnaire respondents to provide a glass of water that would be given to a child (the “point of consumption”) and also visited the main source of water used by the household (the “point of collection”). Water was tested in the households using a portable testing kit based on the EZ-Fit system (Millipore), where 100 ml of water was filtered through a nitrocellulose membrane and then placed onto a selective enzymatic growth media for *E*. *coli* (Nissui Compact Dry EC).

The plates were incubated for 24 to 36 hours, first at body temperature (while in the field) and then overnight in an NQ09 incubator (Darwin Chambers) at 35°C to allow bacterial colonies to grow. After this time period, the number of *E*. *coli* colonies were counted, recorded and merged into the survey micro dataset. We calculated risk levels for *E*. *coli* based on the number of colonies counted in 100 ml of water: <1 (“low risk”), 1–10 (“moderate risk”), 11–100 (“high risk”), >100 (“very high risk”) per 100 ml [21]. Testing details can be found online in the MICS Manual for Water Quality Testing [22].

To measure water availability, we reviewed a number of approaches to questions used in a range of large-scale surveys across different countries [23]. Some approaches focussed only on piped sources which is not applicable to many country contexts while others asked about quantity of water which is difficult to estimate. We focussed our questions on one somewhat subjective measure (WS5C in sample 1) and an objective measure (WS5E in sample 2) and included these in the household questionnaire. The first availability question (WS5C) was selected in order to match as closely as possible the definition of “available when needed” used by the JMP for monitoring safely managed services. We sought to understand whether respondents were unable to meet their domestic drinking water needs and chose a recall period that was longer than that used in DHS (2 weeks). The second question (WS5E) was formulated as a more objective assessment of whether water was always available from the main source of drinking water, with a follow up question to understand the frequency of interruption of supplies. Since occasional interruptions in service are very common in many parts of the world [24]and may not prevent household’s meet their domestic needs we expected a larger proportion of negative responses for the objective assessment of whether water was always available (WSE).

WS5C. Has there been any time in the last month when you have been unable to access water in sufficient quantities when needed? (Sample 1)
Yes.......................1
No........................2
DK........................8
WS5E. Is water always available from your main source of drinking water? (Sample 2)
Yes.......................1
No........................2
DK........................8

The management of onsite sanitation facilities is recognised as a major data gap globally [25]. New questions on emptying of sanitation facilities that are not connected to sewers were devised by WASH and MICS experts to measure when and how pit latrines or septic tanks were emptied. These questions were asked to households reporting the use of pit latrines or septic tanks. Question WS8A was asked to establish whether the onsite facility had been emptied and if so when was the last time. This information is critical to the understanding of the sanitation chain for onsite sanitation facilities. A follow up question was asked to establish who had emptied the sanitation facility, with the aim to understand if the emptying had been conducted by a service provider or by the household. The same questions were used in both samples due to the expected low frequency of emptying and we anticipated emptying by service providers to be the most common approach in Stan Creek.

WS8A. When was the last time your pit latrine or septic tank was emptied?
Probe: How many months/years ago?
Number of months.........................................................1 __ __
Number of years............................................................2 __ __
Never............................................................................995 (SKIP to WS8C)
DK...............................................................................998
Other *(specify)* ____________.....................................996
WS8B. How was it emptied?
Probe: Was it emptied manually or mechanically? Was it emptied by members of your household or by a public or private service provider?
Mechanical
By a household member.............................1
By a service provider.................................2
Manual
By a household member.............................3
By a service provider..................................4
Other *(specify)* ____________..................6
DK.............................................................8

Three new MHM questions developed in consultation with MHM and MICS experts were included as part of the Questionnaire for Individual Women (the same in both samples). Based on WASH definitions of MHM facilities [26], these focused on having a private place for washing during a menstrual period, access to materials for managing a period and disposal of these materials. The first question, UN14, was asked to understand whether women had a private place to change and a follow up probe established whether this was the toilet/bathroom. The second and third questions related to access to materials for managing menstruation and, where required, their disposal. Women responding that they had had a period in the last year were asked this set of questions.

UN14. Did you have a private place to wash yourself when needed during your last menstrual period?
Yes, in the toilet or bathroom........................1
Yes, elsewhere..............................................2
No................................................................3
DK................................................................8
UN15. Did you have access to materials when needed for managing your last menstrual period?
Yes, (disposable after each use).............................1
Yes, (both disposable and re-usable)......................2
Yes, (reusable).......................................................3 (SKIP to next module)
No access to materials............................................4 (SKIP to next module)
No, do not use materials.........................................5 (SKIP to next module)
DK........................................................................8 (SKIP to next module)
UN16. How do you usually dispose of materials after use?
In a special bin.......................................................01
In a bin with other household waste........................02
In the latrine...........................................................03
Burning..................................................................04
Burying..................................................................05
In a field, bush, water body, beach
or other open space...............................................06
Other (*specify*)________......................................96
DK........................................................................98

#### Behaviour coding of interviews

We devised a real-time behaviour coding technique to identify questions with systematic deviations from an ideal question-response process. Behaviour coding, though usually done by observers, was modified to self-coding by interviewers for pragmatic reasons. After each question for which behaviour coding was assigned, a list of behaviour codes appeared on the tablet screen, from which the interviewer selected the codes corresponding to what occurred during the interview. Codes referred to interviewer and respondent behaviours, which is standard practice [27]. The codes noted if the interviewer modified or repeated the question and whether she probed. Respondent codes included whether the respondent interrupted the interviewer, asked for repeats or clarifications, expressed uncertainty about the answer, or gave an answer that was difficult or impossible to code.

### Qualitative approach

We conducted cognitive interviews of respondents and focus groups with interviewers.

#### Cognitive interviews of respondents

We conducted cognitive interviews with a purposive subset of survey respondents to qualitatively evaluate question quality. In total, we conducted 17 cognitive interviews with respondents during and immediately following question administration, while in the field. The aim of the cognitive interviews of respondents was to analyse their understanding of questions and question wordings, of item-specific objectives and ancillary item-specific instructions. We aimed to explore how good an indicator particular questions were for the concepts surveyed, what their (potential) problems were, and how to mitigate those. The interview process used a standardized approach asking respondents to describe how they got to their answer, followed by prompts such as “Think aloud” and probes such as “Why did you give the answer you gave?”, “Why did you answer yes/no? Why not otherwise?” Participants were a convenience sample of respondents. We trained interviewers to purposely identify survey respondents who were insightful about the issues surveyed and who were willing and had time to further discuss these items in detail. All participants provided informed consent to the cognitive interview. Interviews were conducted by a qualitative scientist hired by the MICS team. All interviews followed a semi-structured questionnaire, were audio-recorded and transcribed verbatim.

#### Focus groups of interviewers

At the end of data collection, we conducted two focus groups, one with each survey team, consisting of 5 interviewers and a supervisor each. We developed a semi-structured focus group guide to examine usability of water quality testing protocols, describe how respondents reacted and responded to WASH questions and difficulties that interviewers had in implementing the household survey. The focus group discussion was moderated by a qualitative scientist and aided by an assistant who took detailed notes of discussion points and quotations.

### Analysis

We analysed survey data using univariate and bivariate statistics with STATA 14, weighting data for non-response. Using a content analysis approach, we examined qualitative text data using Nvivo 10, simultaneously reviewing frequencies of the behaviour coding for information on question comprehension and response processes in addition to the qualitative interview text data.

## Results

### Characteristics of households and women

Table 1 shows the characteristics of the households that were interviewed using the household survey. Overall, there sample was somewhat more rural than urban. A large majority of the heads of household were male. Three in four households had piped water while close to one in five relied on packaged water for drinking. The majority (69%) had a toilet facility that flushed to a septic tank while close to 20 percent relied on an improved latrine (see Table 1).

**Table 1:** Characteristics of households interviewed in the household survey.

	Percent	N
Total	100.0	429
Sample		
1	51.7	222
2	48.3	207
Area		
Urban	42.7	183
Rural	57.3	246
Sex of head of household		
Male	73.6	316
Female	26.4	113
Source of drinking water		
Piped	75.0	322
Packaged water	19.4	83
Rainwater collection	3.9	17
Unimproved	1.7	7
Type of sanitation facility		
Flush to sewer	1.1	5
Flush to septic tank	69.1	296
Improved latrines	18.7	80
Unimproved	11.0	47

In total, 267 women were interviewed for the household survey, with slightly more in sample 1 (57 percent) than sample 2 (43 percent, see Table 2). The majority were from rural areas (55 percent), married (66 percent) and had secondary or higher education (55 percent).

**Table 2:** Characteristics of women in the household survey.

	Percent	N
Total	100.0	267
Sample		
1	57.0	152
2	43.0	115
Area		
Urban	44.9	120
Rural	55.1	147
Age		
Mean	27.9	267
Std. deviation	9.2	
Marital status		
Currently married/in union	66.4	177
Widowed	0.9	2
Divorced	0.3	1
Separated	7.8	21
Never married/in union	24.6	65
Education		
None	0.4	1
Primary	45.0	120
Secondary	33.2	89
Higher	21.3	57

Respondents to the cognitive interviews were 24 percent male and 76 percent female. They ranged in age from 18 to 65. About 41 percent lived in an urban area and 59 percent in a rural area. Cognitive interviews lasted a median of 28 minutes (range: 12–58 minutes). We included text data from the shorter interviews in the analysis to the extent that they provided information on question quality.

### Testing water for *E*. *coli* in households: Respondent and interviewer experiences

Overall, respondents readily accepted to have their water tested for *E*. *coli*. All desired test results to be communicated to them, often citing that they were important to the health of their families. One respondent pointed out:

“Well, we would like to know the results for our health and the children’s health.” (Seine Bight, 22 year old female)

When respondents were informed that they would not receive results, many expressed their discontent. The below statements underlie these feelings:

“What? But I need to know if I am drinking healthy water or unhealthy water.” (Dangriga, 40 year old female)

“How would the results benefit me if I do not know what they are?” (Dangriga, 48 year old male)

That they did not receive results was confusing to respondents. Several believed that since they did not receive the results, the water was of sufficiently high quality while in fact *E*. *coli* was later found in some samples.

Data collectors concluded that testing the water in the household was feasible but that finding a flat work space on which to conduct the test proved challenging in some households Further, the interviewers noted that testing water inside the households impedes the questioning process, “because it breaks the momentum of the interview” (Focus group 2). It was therefore agreed that samples collected at the household could be tested immediately after completing the interview (no later than 30 minutes) on the truck bed of the vehicle used to transport enumerators to the field.

### Questions on water availability

The two questions produced similar results; in sample 1, 12 percent of households did not have water at any time in the last month while in sample 2, 10 percent of households said that water was not always available. However, behaviour coding data showed that the first formulation (WS5C) consistently had higher percentages of interviewer and respondent problems (see Table 3), indicating more difficulty to implement WS5C. For example, in the first formulation of the question, close to 25 percent of cases needed further clarification or probing by the interviewer while this occurred in 15 percent of cases in the second formulation of question.

**Table 3:** Summary of behaviour coding.

	Interviewer:		Respondent:					
Question	Modified question	Provided clarification or probed	Asked for repeat	Asked for clarification	Gave answer difficult to code	Expressed uncertainty	Any code	N
**Drinking water availability**								
Water available when needed last month (WS5C)	6.7	23.3	1.8	8.1	1.8	3.1	26.9	223
Water always available (WS5E)	3.9	15.0	1.0	1.9	1.5	1.5	17.5	206
**Emptying of onsite sanitation**								
Last time emptied (WS8A)	6.9	25.7	1.2	5.6	8.8	4.2	30.6	408
How emptied (WS8B)	4.6	18.9	1.0	5.1	4.6	1.0	20.4	196
**Menstrual hygiene management**								
Private place to wash and change (UN14)	9.4	39.0	1.5	8.2	7.1	4.9	43.4	267
Materials for MHM (UN15)	13.9	51.7	1.5	13.1	11.6	5.2	55.8	267
Disposal of materials for MHM (UN16)	6.1	37.6	0.8	5.3	6.8	4.6	38.8	263

Respondents tended to correctly interpret “unable to access water in sufficient quantities when needed” (WS5C) as an interruption in the piping system or insufficient water pressure, as shown in this discourse:

“I: When she asked if you have enough drinking water, how do you come up with your answer of what is sufficient water?

R: If we have enough to drink, cook, clean, bathe.

I: And when she asked about not enough, how did you make the determination?

R: When your water system isn’t working properly or low water pressure, then you can say you do not have enough water to cook.

I: Even when you have water, sometimes it might not be enough.

R: Yes, say, for example, you need to do laundry. The water pressure is too low so it does not full up the machine enough to wash. Same if you need to bathe.” (Dangriga, 28 year old female)

### Measuring the SDG indicator of safely managed drinking water

While virtually all household members used an improved source of drinking water or had drinking water located on their premises (98 and >99 percent respectively), fewer had water available when needed (87 percent) and only about two in three had water free from E. coli (64 percent, see Table 4). These sub-indicators, when combined, show that 57 percent of household members had a safely managed water service, in sharp contrast to the near universal level given by the MDG indicator (98 percent). By type of water source, the SDG value differed, with packaged water scoring higher than other sources, because of better water quality (see Table 4). Expected patterns of lower coverage of safely managed drinking water services among rural households are noted though patterns by wealth are not clear.

**Table 4:** Safely managed drinking water services in Stan Creek, Belize ^a^.

	Proportion of household members:					
	Using an improved source of drinking water (MDG 7.C)	With drinking water available when needed	With drinking water on premises	Without *E*. *coli* in drinking water collected at source	Safely managed drinking water services (SDG 6.1^b^^)^	Number of household members
**Total**	**98.0**	**87.3**	**99.9**	**64.1**	**57.1**	**1133**
**Water source**						
Piped water	100.0	86.1	100.0	61.8	54.9	922
Packaged water	100.0	89.9	100.0	87.8	77.7	165
Other	51.1	100.0	97.6	26.8	26.8	47
**Residence**						
Urban	100.0	100.0	83.4	85.9	71.6	438
Rural	96.7	99.8	89.7	50.3	47.9	695
**Household wealth**						
Poorest	94.3	86.6	99.5	43.5	40.4	221
Second	100.0	90.1	100.0	56.3	54.3	228
Third	95.6	93.6	100.0	67.0	62.1	231
Fourth	100.0	87.4	100.0	82.8	73.5	239
Richest	100.0	77.8	100.0	69.9	53.4	214

^b^ Proportion of household members with an improved source of drinking water, with drinking water available when needed, with drinking water on premises and household without *E*.*coli* in drinking water collected at the source

^a^ Results shown for households with with a valid *E*.*coli* result

**Fig 1:**
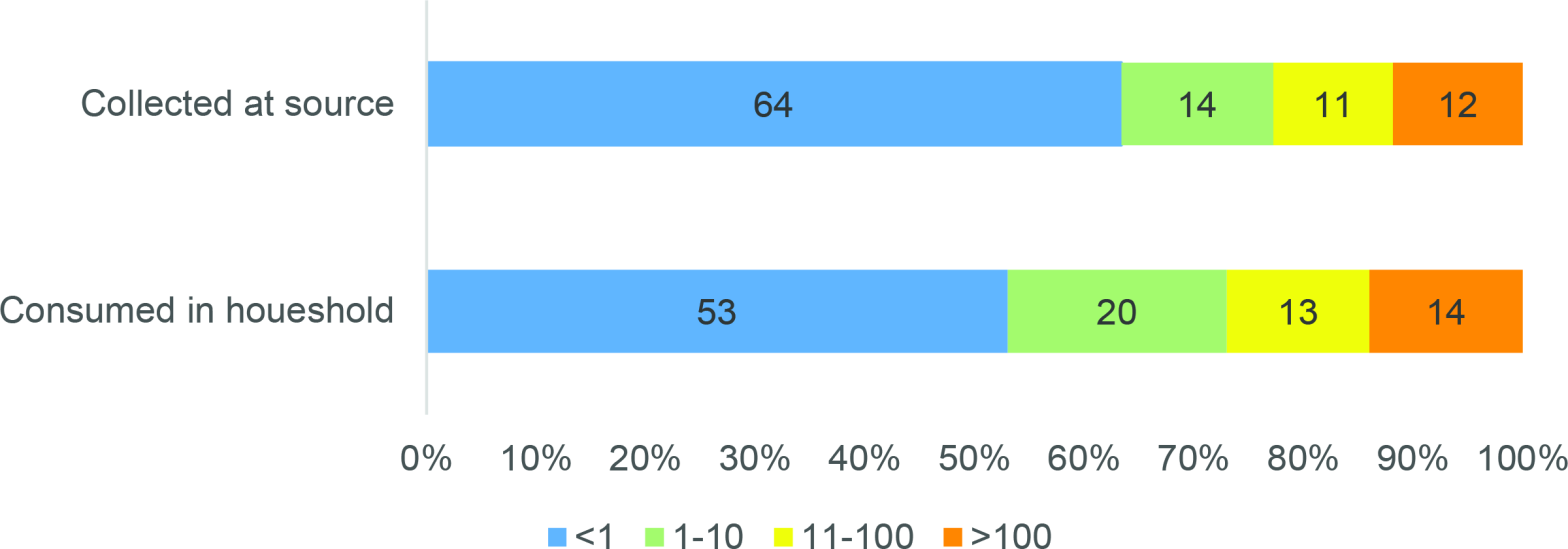
Levels of E. coli in drinking water collected at the source and consumed in the household.

The proportion of samples with detectable *E*. *coli* levels in drinking water increased between the point of collection and the point of consumption in the household (Fig 1). Overall, one third (36 percent) of household members collected water from a drinking water source with detectable *E*. *coli* compared with almost half (47 percent) at the point of consumption. The difference was smaller for higher levels of *E*. *coli*: 23 percent of samples were over 10 *E*. *coli* per 100 mL at the point of collection compared with 27 percent at the point of consumption. Contamination was higher in piped than packaged water, and much higher in ‘other sources’ (Table 4).

### Emptying of pit latrines and septic tanks

Table 5 shows that a majority of pit latrines and septic tanks had never been emptied (53 percent), and about 20 percent were emptied in the previous five years. About one in four respondents did not know whether their facility had been emptied or could not provide an answer. The non-response to the question on “the last time emptied” (WS8A) is partly explained by qualitative findings. Interviewers in one focus group identified that respondents found the question inapplicable to pit latrines as the usual practice is to cover the hole when it is full and make a new one. Respondents renting accommodation also did not know when the septic tank was last emptied as this is the responsibility of the landlord. Behaviour coding indicated that in about one quarter of the cases, the interviewer had to provide clarification or probe further (see Table 3). We observed little to no heaping of data on expected categories in the distribution of the data on timing of emptying.

**Table 5:** Emptying of sanitation facilities in households with latrines or septic tanks.

	Time last emptied						Means of emptying among households where emptying occurred				
	<1 year	1–4 years	5+ years	DK/ Miss.^a^	Never emptied	N	Mechanical by a service provider	Manual by household member	Manual by a service provider	DK/ Miss.	N
**Total**	**8.8**	**11.4**	**2.6**	**23.5**	**53.4**	**405**	**92.7**	**1.2**	**2.3**	**3.3**	**92**
Area											
Urban	9.5	20.1	3.4	25.0	42.0	173	94.2	0.0	4.5	1.4	57
Rural	8.3	4.9	1.9	22.4	62.5	232	90.3	3.2	0.0	6.5	35
Type of sanitation facility											
Septic tank	8.6	15.1	3.1	27.4	45.9	300	95.5	0.0	2.1	2.4	80
Latrine	9.4	0.8	1.1	12.6	76.1	105	73.7	9.5	7.3	9.5	12

^a^DK/Miss: don’t know or missing responses

In households where respondents reported that the sanitation facility had been emptied, the vast majority had been emptied mechanically by a service provider (see Table 5). Behaviour coding showed that in about 20 percent of cases, additional interviewer probing and clarification was provided, and in about 5 percent of cases the interviewers modified the question.

### Menstrual hygiene management

Virtually all women age 15–49 interviewed had a private place to wash (95 percent), nearly always referring to the toilet or bathroom, and nearly all had access to materials for managing their periods (98 percent). About half of women used a ‘special bin with a lid’ to dispose of the menstrual materials, while 14 percent disposed of the materials in a latrine and 16 percent burned the materials. Rural women were more likely to dispose of menstrual materials in a latrine or burn the materials than urban women (see Table 6).

**Table 6:** Menstrual hygiene management.

	Total	Area	
		Urban	Rural
**Number of women**	**267**	**120**	**147**
Private place to wash (%)			
Yes, in the toilet or bathroom	91.5	97.8	87.3
Yes, elsewhere	3.3	1.4	4.9
No	4.0	0.0	7.3
Missing	1.2	0.8	1.6
Materials for managing period (%)			
Yes, disposable	98.3	99.2	97.6
No access to materials	0.4	0.0	0.8
Missing	1.2	0.8	1.6
Place for disposal of menstrual materials (%)	**263**	**119**	**143**
In a special bin	52.3	74.7	33.8
In a bin with other household waste	8.5	14.9	3.3
In the latrine	16.8	4.2	27.3
Burning	14.5	4.1	23.2
Burying	2.7	0.0	5.0
In a field, bush, water body, beach or other open space	3.8	1.4	5.8
Other	1.2	0.7	1.7

In general, these questions were acceptable as part of the household survey; few women refused to answer these questions (less than 2 percent) and women, during cognitive interviews, showed few inhibitions discussing MHM with the exclusively female interviewers.

Behaviour coding, however, indicated that in 38 to 52 percent of cases for the three question, interviewers needed to clarify the questions or probe to elicit responses. Of the three questions, UN15 (on access to materials for the period) posed the greatest challenge to the interviewers in terms of clarifications and probing. Cognitive interviews provide further data on this. Words such as “washing” in UN14 evoked from some women, the idea of a bath tub. However, for most women interviewed, this could only mean a bathroom. The term ‘private’ seemed to cause some confusion as this respondent explains her thought process,

“Well, that one [referring to “private”] was a little strange, but I just think about the norm like the bathroom. When I hear private, I say all people will be the same, because private means, it will be just you. When I hear private place, it sounds like something other than the bathroom. It made it seem like you have other options.” (Dangriga, 28 year old female)

Still other women thought that “private place”, because it can only be a bathroom for menstrual hygiene, was a private room or bathroom exclusively for one individual woman to use:

“I told her no, because we only have one bathroom that everyone uses.” (Pamona, 40 year old female)

The focus groups also pointed out that the term “materials” (UN15) was not well-understood, in agreement to the behaviour coding. One focus group cites,

“The women did not understand that question. When the women hear materials, they think about materials to wash.” (Focus Group 1).

All women interviewed saw no problem with disposal of materials (UN16). They described the various ways of disposing, from wrapping in toilet paper, to placing in trash to burning. Interviewers concurred, citing that women often provided an elaborate response of how they disposed,

“They gave us a full briefing of how they disposed.” (Focus Group 1)

However, consistent with the high levels of probing seen in the behaviour coding (38 percent), most interviewers agreed that the place of disposal prompted considerable probing.

## Discussion

This study evaluated new protocols and questions for enhancing WASH monitoring in the MICS surveys in order to track progress towards the SDG targets for drinking water, sanitation and hygiene. This is one of the few studies to examine new protocols on WASH monitoring for the SDGs using household surveys. The study tested different questionnaire approaches and protocols and indicated that some specific refinements to these are needed for the next round of MICS surveys.

The study found that drinking water consumed in the household was somewhat more likely to be contaminated than water collected at the source, similar to other studies of contamination of drinking water after collection from the main source [28,29]. It also highlights that the proportion of population using sources which meet the new SDG criteria for ‘safely managed’ drinking water is likely to be substantially lower than estimates based on the old MDG indicators [30]. These results underscore the value of indicators which go beyond access to infrastructure and reveal inequalities in service levels.

Respondents understandably want to know the results of water quality tests and future survey should clearly explain to respondents whether or not they will receive test results. Logistically, this is difficult as results are currently available 24 hours or later after the sample has been processed, at which time, field teams may have already left the area. Returning results also raises additional questions: does a single test provide enough evidence to indicate the need to change water treatment and storage practices? Are results enough for households to change behaviours? Or are governments better able to act on the results at the aggregate level? The Medical Research Council (UK) Wellcome Trust points out that further evidence on the utility of returning results on health-related findings is still needed [31]. We also consider that in many countries, providing drinking water is the responsibility of governments, which are better placed to identify and address sources of contamination than household surveys which can provide only minimal information to households safe handling and storage. The MICS programme recommends that national governments who own and implement the MICS surveys devise strategies for disseminating results to fit the needs of their contexts.

Testing water inside households interrupted the flow of the interview and increased the time in the household. Based on this finding, in the current round of MICS, we propose that field teams collect water samples and immediately perform testing after leaving the household. We also note that we tested water in all households to boost the sample size for analysis. In actual MICS surveys, countries are advised to test water in 3–5 households per cluster, which reduces the overall implementation cost for equipment and time in the field. Our results also showed that water availability can be measured in MICS, as is done in other household surveys [23]. Though the subjective measure produces somewhat more difficulty for interviewers and respondents, respondents were able to understand key elements of the question, such as having sufficient water and having it when needed, which is essential to having a broader understanding of the issue. For the latest round of MICS, we include this subjective measure and will closely monitor its performance across countries.

Safely managed sanitation services represent an ambitious new benchmark for global monitoring and tracking progress requires data from both household surveys and regulatory authorities or service providers [32]. In order for services to be considered safely managed excreta must be safely disposed of in situ or transported and treated offsite([25]. This study sought to understand the extent to which information for onsite sanitation facilities could be captured in MICS surveys–we focused on emptying of onsite sanitation facilities which is the first step in the faecal sludge management chain [33] and who was responsible for emptying the facilities. In Stann Creek, Belize, emptying of these facilities is not prevalent, and when done, it is performed mechanically by a service provider. This poses important questions about how the emptying is conducted and whether these service providers are treating the wastes appropriately or disposing of them inappropriately. A study of 12 cities suggests that in many settings faecal sludge is not being managed effectively [34]. We found significant numbers of respondents were not aware of emptying practices and this may be partly due to the fact that the person interviewed may not be responsible for construction and maintenance of sanitation facilities. This lack of knowledge poses challenges for monitoring emptying practices through household surveys and in itself may reflect systems that are not safely “containing” human waste. While MICS6 has adopted these questions as part of routine data collection and added a further question to attempt to establish where the excreta are disposed to (see MICS6 questionnaires at mics.unicef.org/tools), further exploratory work on emptying in settings with higher prevalence and more varied practices is planned for several countries as part of the Global Expanded Monitoring Initiative [35]. Moreover, there is a need to establish approaches for the collection of data on the rest of the faecal sludge management chain (transport, treatment, disposal/reuse).

The ability to manage menstruation safely and with dignity is fundamental for achieving gender equality [36]. A recent UN Women discussion paper describes many of the challenges women and adolescent girls face–ranging from lack of privacy and knowledge to ingrained taboos and social restrictions [37]. In consultation with MHM experts, we selected a short set of questions to examine whether women and girls (15–49) were able to wash in private during their last period, had access to menstrual materials and a suitable location to dispose of these. Only a small minority of women and girls in this study had difficulties with these issues, and this contrasts sharply with other studies including in India and Kenya, where access to materials, disposal and privacy for washing was much less common [38,39]. While some terms in the MHM questions were difficult to understand for respondents, we show that discussion of this topic was acceptable in a household survey in Stann Creek, Belize. To improve understanding of the concepts, MICS6 questions focus on being able to wash and change in privacy (rather than asking about a ‘private place’) and list types of menstrual hygiene management materials rather than referring to ‘absorbent materials’. These data collected in MICS6 will be the first global attempt to quantify the challenge that women and girls have to manage menstruation safely and with dignity and will complement information collected on facilities at institutional settings, including schools [40].

This study has several limitations. The survey data are representative of the Stann Creek district, Belize. As MICS surveys are conducted in a wide variety of contexts, we may not have identified all challenges with the new topics. WASH services in Stann Creek are better than many parts of the world, limiting the generalisability of the lessons learnt from this pilot. In particular, water was found to be available in almost all cases and most women had access to improved sanitation and used disposable materials. Further work is needed to ensure that the questions are appropriate for other contexts and understood by respondents in these contexts. Survey programmes are encouraged to “pre-test” questionnaires, an approach that is always applied in MICS surveys. The cognitive interviews and two focus groups covered a wide range of topics new to MICS. With limited numbers, the study may not have reached saturation on every topic explored. Nevertheless, patterns across the various evaluation techniques provided coherent, logical and explainable patterns. We advocate for a continued agenda of testing survey questions across different settings which would ultimately build a more comprehensive body of evidence on the performance of survey questions and protocols.

Collectively, these findings have been used to improve the protocols and guidelines of the MICS programme (available at mics.unicef.org/tools) which supports roughly 50 to 60 countries per round and covers all regions of the world. These are also useful to inform measurement guidelines from the WHO/UNICEF JMP and can potentially be adapted and adopted by other household survey programmes such as the DHS and LSMS. These actions will ultimately contribute to increasing the availability of more harmonized and comparable data for monitoring the new elements of SDG targets 6.1 and 6.2 at the country and global levels.

## Supporting information

S1 File Water quality data. (XLSX)

S2 File Household and WASH data. (XLSX)

S3 File Individual women data. (XLSX)

S4 File Behaviour coding data. (XLSX)
